# Rat-Bite Fever in a 34-Year-Old Female

**DOI:** 10.7759/cureus.42453

**Published:** 2023-07-25

**Authors:** Nada Mohamed, Said Albahra, Christopher Haley

**Affiliations:** 1 Pathology, Baylor Scott & White Medical Center - Temple, Temple, USA; 2 Dermatology, Baylor Scott & White Medical Center - Temple, Temple, USA

**Keywords:** leukocytoclastic vasculitis, rash, fever, streptobacillus moniliformis, rat-bite fever

## Abstract

Rat-bite fever (RBF) is a rare systemic infectious disease caused by *Streptobacillus moniliformis*, *Spirillum minus*, or *Streptobacillus notomytis*. As the name implies, the disease is typically transmitted by a rat bite. RBF usually presents as a combination of fever, arthritis, and rash. Definitive diagnosis of RBF may prove difficult, as the responsible bacteria are not easily identified with standard testing. We describe a case of RBF in a 34-year-old female who presented with fever, chills, polyarthralgia, and skin rash following a rat bite. Initial vital signs were remarkable for fever and tachycardia. Physical examination revealed an erythematous vesicular and papular rash involving her extremities, buttocks, and oral mucosa. Blood cultures were negative. A skin biopsy revealed leukocytoclastic vasculitis and was negative for Gram stain. Further analysis using specialized immunohistochemistry and polymerase chain reaction (PCR) identified *S. moniliformis*. A diagnosis of RBF was made, and the patient was successfully treated with a two-week course of doxycycline.

## Introduction

Rat-bite fever (RBF) is a rare zoonotic illness caused by *Streptobacillus moniliformis, Spirillum minus, *or* Streptobacillus notomytis. S. moniliformis*, a fastidious gram-negative bacillus, is the most common cause of RBF in North America and Europe, whereas *S. minus* and *S. notomytis* infections are more prevalent in Asia [1,2]. The disease is transmitted through rodent bites or consumption of contaminated food or water, with an average incubation period of five days [3,4]. RBF typically presents as a triad of fever, arthritis, and rash, with the morphology of the rash being highly variable. If left untreated, RBF can lead to severe complications and even death [5,6]. Confirming a diagnosis of RBF can be challenging. The inert nature of the culprit bacteria often makes routine blood or tissue cultures inadequate to support its growth. Additionally, direct specimen testing by Gram staining may produce negative results. Therefore, specialized techniques such as immunohistochemistry, polymerase chain reaction (PCR), or rRNA sequencing may be necessary to ensure an accurate diagnosis [1,7].

## Case presentation

**Figure 1:**
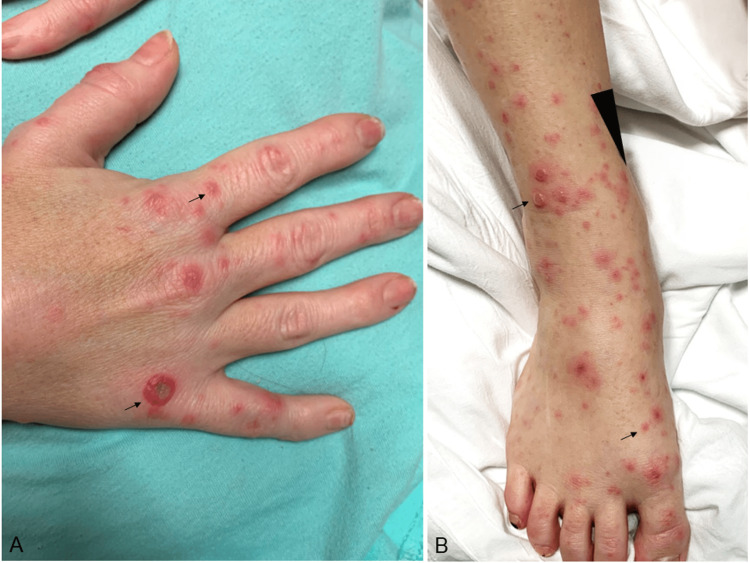
Erythematous vesicular and papular rash on the hand dorsum (A) and the extensor surface of the leg and dorsal feet (B)

A 34-year-old woman presented with a two-day history of malaise, fever, chills, and polyarthralgia and a one-day history of tender, slightly pruritic rash of the extremities and oral mucosa. The symptoms began two days after she was bitten by a rat she was feeding to her pet anaconda. The patient was allergic to penicillin and had a history of asthma, gastroesophageal reflux disease, migraines, and an aborted cerebrovascular accident. Blood pressure was 111/68 mm Hg, heart rate was 115/min, respiratory rate was 18/min, temperature was 100 °F, and pulse oximetry was 98%. Physical examination revealed an erythematous vesicular and papular rash involving the extensor surface of her extremities, buttocks, mucosal lower lip, and tongue (Figure 1).

**Figure 2:**
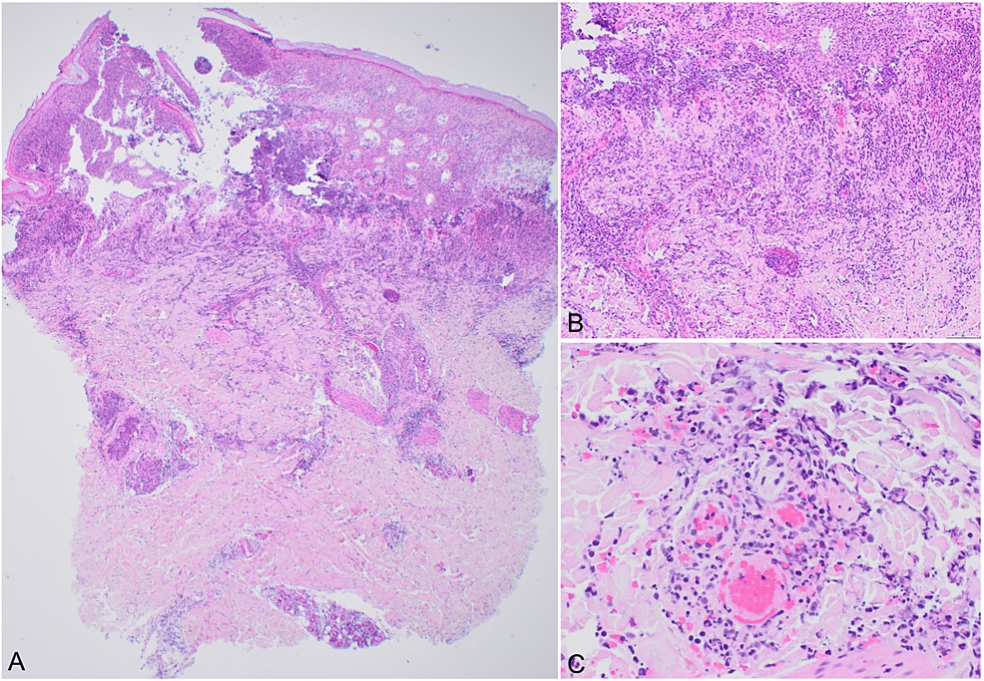
Histologic changes of the skin biopsy (A) Prominent superficial and deep perivascular mixed inflammatory infiltrate with subepidermal clefting (x20). (B) Superficial dermal congestion and dense neutrophilic infiltrate extending to the epidermis (x100). (C) Leukocytoclastic vasculitis with perivascular neutrophilic infiltrate with necrosis and fibrin thrombi (x400).

Complete blood count showed white blood cells of 9.4 x 10^9^/L, platelets of 143 x 10^9^/L, and hemoglobin of 11.9 g/dL. Tests for chlamydia, gonorrhea, HIV, and syphilis were all negative. Due to a high clinical suspicion of RBF, the patient was immediately started on intravenous ceftriaxone after blood cultures were drawn. However, the blood cultures ultimately returned negative. A punch biopsy from the left elbow revealed a prominent superficial and deep dermal leukocytoclastic vasculitis with necrosis and fibrin thrombi. The superficial dermis displayed congestion, edema, erythrocyte extravasation, and neutrophilic inflammation extending into the epidermis, with subepidermal clefting (Figure 2).

Gram stain of the biopsy was negative. The histologic findings were concerning for septic vasculitis, and the tissue was sent to the Centers for Disease Control and Prevention for further workup. Tissue sequence analysis of 16S rRNA gene PCR assay and immunohistochemistry were both positive for *S. moniliformis*, confirming a diagnosis of RBF. Our patient was seen for a follow-up 2.5 weeks after the onset of her symptoms. She had completed a two-week course of doxycycline, as she was penicillin-allergic. Her malaise, fever, chills, polyarthralgia, cutaneous lesions, and oral lesions had all resolved without issue.

## Discussion

*S. moniliformis* is the primary causative organism of RBF in North America and Europe [1]. It is a facultatively anaerobic gram-negative rod that resides as normal flora in the upper respiratory tract of pet, wild, and laboratory rodents [5,7]. The transmission of RBF commonly occurs after a bite from a rat or other rodents; however, it may also occur through handling of rodents, exposure to rodent excreta or saliva, or human ingestion of rodent-contaminated food or water [8]. In the majority of reported RBF cases, rodent exposure without a known bite was reported, while actual rat bites, such as in our case, were reported in less than half of cases [2,9].

The most common presentation of RBF is a triad of fever, polyarthritis (often migratory and more prominent on lower extremities), and rash beginning 3-10 days following rodent exposure [1,10]. Similar to our case, reported cases of RBF have demonstrated the presence of at least two symptoms out of the typical triad [2,5-7,10-16]. Additional symptoms, such as lymphadenopathy, chills, headache, malaise, nausea, vomiting, and myalgias, have also been reported [1,8]. The cutaneous manifestations of RBF are variable and may include morbilliform, vesicular, pustular, petechial, or purpuric lesions. The extensor extremities are most commonly affected, followed by the palms and soles [2,11]. Mucosal involvement was less commonly reported in the literature [2]. Histopathological analysis of cutaneous lesions in previously reported cases revealed findings consistent with our case, including leukocytoclastic vasculitis and neutrophilic inflammation [6,12-14]. It is worth noting that Gram stain revealed the organism in only approximately half of the cases and negative Gram stains should not be used to rule out RBF [6,13]. Leukocytosis and elevated inflammatory markers were commonly observed in reported cases of RBF, similar to our findings [2,12,16]. Occasionally liver enzymes can also be elevated [12,15]. While positive blood cultures served as the basis for diagnosis in many cases, a significant proportion of cases, including our case, yielded negative blood cultures [12,16-18]. The microbiology laboratory should be notified if RBF is suspected as S. moniliformis is a fastidious organism that grows slowly and requires microaerophilic conditions and special and enriched media for culture [4]. In such instances, the diagnosis required PCR and/or rRNA sequencing, which is usually more sensitive than culture or Gram stain [12,16].

Prompt diagnosis and treatment of RBF are imperative, as delays in treatment may lead to serious complications including endocarditis, pericarditis, interstitial pneumonia, hepatitis, nephritis, meningitis, brain abscess, serositis, systemic vasculitis, discitis, and sepsis [2,9,12,16]. Overall mortality in untreated patients may be as high as 13%, with endocarditis being the leading cause of death [2,5,6,10]. Despite being a gram-negative bacteria,* S. moniliformis* is susceptible to most gram-positive antimicrobial agents such as penicillins, tetracyclines, cephalosporins, clindamycin, macrolides, and aztreonam [1,19] and is intermediately susceptible to the aminoglycosides and fluoroquinolones. Intravenous penicillin is usually the first-line treatment, with tetracycline, doxycycline, and streptomycin serving as alternatives for penicillin-allergic patients [10]. In most reported RBF cases, antibiotic treatment for 1-2 weeks is sufficient for disease resolution. Longer treatment duration may be required if systemic RBF complications occur. Systemic corticosteroids have been utilized in some cases, but their role is unclear given their immunosuppressive nature.

## Conclusions

In conclusion, RBF is a rare yet potentially fatal disease that can be easily overlooked due to its nonspecific symptoms. Awareness of this entity and a high level of suspicion based on the patient's history are essential for prompt diagnosis and treatment. Diagnosis of RBF should not be solely reliant on Gram stain or routine blood culture, as false negative results are not uncommon. Histopathological examination typically reveals a prominent leukocytoclastic vasculitis and neutrophilic infiltrate, though these findings are not specific to RBF. Alternative diagnostic methods, such as special immunohistochemical stains, PCR, and/or rRNA sequencing, may be considered to confirm RBF diagnosis.

## References

[1] (2022). Rat Bite Fever (RBF) | CDC. https://www.cdc.gov/rat-bite-fever/index.html.

[2] Kämmerer T, Lesmeister T, Wollenberg A, French LE, Strobel E, Reinholz M (2021). Rat bite fever, a diagnostic challenge: case report and review of 29 cases. J Dtsch Dermatol Ges.

[3] Gaastra W, Boot R, Ho HT, Lipman LJ (2009). Rat bite fever. Vet Microbiol.

[4] Hall GS, Woods GL (2017). Medical bacteriology. Henry’s Clinical Diagnosis and Management by Laboratory Methods.

[5] Cunningham BB, Paller AS, Katz BZ (1998). Rat bite fever in a pet lover. J Am Acad Dermatol.

[6] Miraflor AP, Davallow Ghajar L, Subramaniam S (2015). Rat-bite fever: an uncommon cause of fever and rash in a 9-year-old patient. JAAD Case Rep.

[7] Elliott SP (2007). Rat bite fever and Streptobacillus moniliformis. Clin Microbiol Rev.

[8] Kache PA, Person MK, Seeman SM, McQuiston JR, McCollum J, Traxler RM (2020). Rat-bite fever in the United States: an analysis using multiple national data sources, 2001-2015. Open Forum Infect Dis.

[9] Croker BA, Prudence A, Wilson PA, Givney R, O’Kane G (2021). Rat-bite fever due to Streptobacillus moniliformis: a case series from New South Wales, Australia, and literature review. Infect Dis Clin Pract.

[10] Kwon CW, Somers K, Scott G, Mercurio MG (2016). Rat bite fever presenting as palpable purpura. JAMA Dermatol.

[11] Freels LK, Elliott SP (2004). Rat bite fever: three case reports and a literature review. Clin Pediatr (Phila).

[12] Akter R, Boland P, Daley P, Rahman P, Al Ghanim N (2016). Rat bite fever resembling rheumatoid arthritis. Can J Infect Dis Med Microbiol.

[13] Georgescu V, Lespessailles E, Martin L, Poisson DM, Estève E (2002). Digital purpura revealing septicaemical rat-bite fever (Article in French). Ann Dermatol Venereol.

[14] Kawakami Y, Katayama T, Kishida M, Oda W, Inoue Y (2016). A case of Streptobacillus moniliformis infection with cutaneous leukocytoclastic vasculitis. Acta Med Okayama.

[15] Kasuga K, Sako M, Kasai S, Yoshimoto H, Iihara K, Miura H (2018). Rat bite fever caused by Streptobacillus moniliformis in a cirrhotic patient initially presenting with various systemic features resembling Henoch-Schönlein purpura. Intern Med.

[16] Shadrin IY, Albitar HA, Paim AC, Issa M, Wilson WR (2020). Migratory polyarthralgias and skin rash: rat bite fever with a positive anti-cyclic citrullinated peptide. Mayo Clin Proc Innov Qual Outcomes.

[17] Dworkin J, Bankowski MJ, Wenceslao SM, Young R (2010). A case of septic arthritis from rat-bite dever in Hawai‘i. Hawaii Med J.

[18] Budair B, Goswami K, Dhukaram V (2014). Septic arthritis secondary to rat bite fever: a challenging diagnostic course. BMJ Case Rep.

[19] Edwards R, Finch RG (1986). Characterisation and antibiotic susceptibilities of Streptobacillus moniliformis. J Med Microbiol.

